# Metacognitive knowledge and experience across multiple cognitive domains in euthymic bipolar disorder

**DOI:** 10.1192/j.eurpsy.2021.31

**Published:** 2021-06-04

**Authors:** Ivan J. Torres, Ceren Hidiroglu, Sylvia A. Mackala, Sharon Ahn, Lakshmi N. Yatham, Eysegul Ozerdem, Erin E. Michalak

**Affiliations:** 1 Department of Psychiatry, University of British Columbia, Vancouver, British Columbia, Canada; 2 British Columbia Mental Health and Substance Use Services, Vancouver, British Columbia, Canada; 3 Department of Psychology, Dokuz Eylul University, Izmir, Turkey; 4 Department of Psychiatry and Psychology, Mayo Clinic, Rochester, Minnesota, USA

**Keywords:** cognition, metacognition, neuropsychology, quality of life, psychopathology

## Abstract

**Background:**

Metacognitive knowledge (MK; general awareness of cognitive functioning) and metacognitive experience (ME; awareness of cognitive performance on a specific cognitive task) represent two facets of metacognition that are critical for daily functioning, but are understudied in bipolar disorder. This study was conducted to evaluate MK and ME across multiple cognitive domains in individuals diagnosed with bipolar disorder and unaffected volunteers, and to investigate the association between metacognition and quality of life (QoL).

**Methods:**

Fifty-seven euthymic participants with bipolar disorder and 55 demographically similar unaffected volunteers provided prediction and postdiction ratings of cognitive task performance across multiple cognitive domains. Self-ratings were compared to objective task performance, and indices of MK and ME accuracy were generated and compared between groups. Participants rated QoL on the Quality of Life in Bipolar Disorder Scale (QoL.BD).

**Results:**

Metacognitive inaccuracies in both MK and ME were observed in participants with bipolar disorder, but only in select cognitive domains. Furthermore, most metacognitive inaccuracies involved underestimation of cognitive ability. Metacognitive indices were minimally associated with medication variables and mood symptoms, but several indices were related to QoL.

**Conclusions:**

Individuals with bipolar disorder demonstrate inaccuracies in rating their cognitive functioning and in rating their online cognitive task performance, but only on select cognitive functions. The tendency to underestimate performance may reflect a negative information processing bias characteristic of mood disorders. Metacognitive variables were also predictive of QoL, indicating that further understanding of cognitive self-appraisals in persons with bipolar disorder has significant clinical relevance.

## Introduction

Although it is well established that individuals with bipolar disorder frequently exhibit cognitive problems in memory, attention, and executive functioning [1,2], there is very little understanding of their metacognitive skills. Metacognition broadly refers to knowledge about one’s own cognitive functioning, and one aspect of metacognition concerns an individual’s ability to accurately assess their own cognitive ability or functioning [3]. Metacognitive impairments are clinically relevant, as they can lead to poor decision-making and difficulties in daily life functioning [4–7]. In the context of bipolar disorder, poor awareness into cognitive difficulties can lead individuals to behave inappropriately in certain situations. For example, if cognitive deficits are not sufficiently appreciated, patients may engage in risky, overconfident behaviors that can lead to poor outcomes or compromise safety and functioning. Alternatively, poor awareness of cognitive abilities or strengths could inhibit individuals from engaging in behaviors or actions that might be beneficial or rewarding, thus diminishing quality of life. In addition to bipolar disorder, problems with metacognition and self-awareness are also proposed to play a role in other mental disorders [8–10].

Two components of metacognition include metacognitive knowledge (MK) and metacognitive experience (ME) [3,11,12]. MK refers to a person’s general beliefs and knowledge about their own cognitive functioning, and can be assessed through ratings or questionnaires about their cognitive functions (e.g., “my memory is good,” “I have problems with my attention span,” etc.). In contrast, ME refers to an individual’s subjective rating of their cognitive performance on a recently completed or ongoing “online” task (e.g., “I performed poorly on this memory test”). In a prediction/postdiction paradigm [13,14], MK can be assessed by asking participants to make predictions of how they think they will perform on an upcoming cognitive task, as this requires the person to draw upon their general belief about their own ability in that cognitive domain. In contrast, to assess ME, participants are asked to provide postdiction ratings about their perceived performance on a just-completed task. In the latter case, in addition to relying on general beliefs about their ability, individuals are also drawing on online experience with the task in order to formulate a judgment about their perceived performance. Predictions and postdictions can then be contrasted to objective performance to determine the absolute accuracy of ratings, and whether ratings tend to over-or under-estimate cognitive functioning (see “Methods”).

Identifying problems in MK or ME is important because it can direct interventions toward modifying general or task-related cognitive beliefs, respectively [15]. Although distinct, MK and ME are also interrelated such that one may influence the other [12,16,17]. That is, general beliefs about one’s cognitive abilities may influence judgments about expected performance on a specific task, and perceived performance on a task may in turn help shape general beliefs about one’s cognitive functioning. Because of this reciprocity and the clinical and functional relevance of metacognitive functioning, it is critical to better understand both MK and ME in bipolar disorder.

There has been little research on metacognition in bipolar disorder, but an emerging literature indicates that subjective ratings of cognitive functioning correspond poorly or inconsistently to actual objective cognitive performance, suggesting impaired MK. The poor correspondence has been most frequently studied and observed in global cognitive functioning [18–24], but has also been reported in specific cognitive domains in which patients typically show cognitive deficits—including attention, memory, and executive function [25]. However, because only a limited number of prior studies have included healthy control groups, it has been difficult to ascertain whether these patterns of poor objective/subjective correlation deviate from that observed in healthy individuals. Moreover, because prior studies are correlational, they do not provide further detail about the nature or source of the MK inaccuracy in bipolar disorder, such as whether individuals under- or over-estimate their abilities. One study indicated that patients tended to underestimate their verbal memory skills relative to working memory and executive functions [26]. Beyond this limited research on MK in bipolar disorder, even fewer studies have investigated ME. One recent study showed that postdiction ratings of global cognitive functioning tended to over-estimate bipolar participant’s actual cognitive performance; however, because over-estimation was also observed in healthy controls, the overall findings suggested similar ME in both groups [27].

In a previous study, our group compared the accuracy of MK ratings (predictions of overall cognitive ability) and ME ratings (postdictions of recent memory task performance) in bipolar disorder relative to control volunteers [15]. Patient predictions of cognitive functioning were inaccurate (impaired MK) and characterized by both over- and under-estimation, but their postdiction ratings of recent memory performance were accurate (intact ME). However, this past study was limited by several factors. First, the MK and ME tasks were confounded by the fact that participants were rating different cognitive domains for each. Thus, rather than showing MK relative to ME deficits, an alternative interpretation of findings could be that participants with bipolar disorder were poor raters of general cognitive skill but not of memory skill. A better test of the MK versus ME deficit hypothesis would require individuals to rate the same cognitive ability for both MK and ME tasks. Another limitation was that only a single measure of MK and ME was used, so it was not possible to assess whether any metacognitive problems might be global or domain specific. Finally, without obtaining prediction–postdiction ratings on the same cognitive ability, it was not possible to evaluate the degree to which accuracy of ratings might be influenced by experience with or exposure to a cognitive task. For example, it might be hypothesized that individual’s ratings might change or become more accurate after experience with a task [28,29], and this could only be determined by comparing predictions and postdictions on the same ability.

In light of these gaps in knowledge, the purpose of the present study was to employ the prediction/postdiction methodology to evaluate whether MK and ME are impaired across multiple cognitive domains in euthymic individuals with bipolar disorder relative to control volunteers. Moreover, if inaccuracies were detected, a further goal was to determine whether individuals with bipolar disorder over- or under-estimate their cognitive functioning. In addition, given that cognitive functioning has been associated with quality of life (QoL) in bipolar disorder [30,31], we sought to evaluate whether metacognitive variables may also associate with QoL. Based on prior work [15,25,26], we hypothesized that participants with bipolar disorder would have more deficits in MK than ME, that metacognitive impairments would be most prominent in memory and executive domains, and that metacognitive variables would predict QoL.

## Method

### Participants

The study was carried out within the Mood Disorders Centre in the Department of Psychiatry, University of British Columbia. Individuals with bipolar disorder were initially recruited from multiple sources including outpatient and inpatient hospital clinics, regional mood disorder groups and associations, local community mental health centers, and through online advertisements. Inclusion criteria were as follows: (a) met The Diagnostic and Statistical Manual of Mental Disorders (DSM)-IV/5 criteria for bipolar disorder I or II; (b) aged 17 or older; (c) euthymic mood based on scores of 8 or lower on the Hamilton Depression Rating Scale [32] and the Young Mania Rating Scale [33]; and (d) fluent in English (based on a self-rating of the person’s ability to speak, read, write, and understand English). Exclusion criteria included (a) history of serious neurological disorder or brain injury and (b) alcohol or substance use in the past month. All participants were stable outpatients at the time of study, and underwent a comprehensive clinical assessment that included documentation of clinical history, symptom ratings, structured clinical interview, and other clinical measures according to a standardized protocol. All participants had been diagnosed with bipolar disorder by a psychiatrist or physician, which was confirmed by both a clinical psychologist and trained research assistant using the Mini-International Neuropsychiatric Interview (MINI) [34].

A comparison group of unaffected volunteers was recruited from the community through online and other community postings. Healthy volunteers were required to be aged 17 or older and to be fluent in English, and were excluded if they had a history of serious neurological disorder or brain injury, history of psychiatric disorder, history of diagnosed psychiatric disorder in first-degree relatives, or history of alcohol or substance use in the past month. Volunteers were also screened using the MINI for presence of psychiatric disorders. Ethics approval was obtained from the University of British Columbia Clinical Research Ethics Board, and all participants provided written informed consent prior to participation. The authors assert that all procedures contributing to this work comply with the ethical standards of the relevant national and institutional committees on human experimentation and with the Helsinki Declaration of 1975, as revised in 2008.

#### Neuropsychological measures

Participants with bipolar disorder and healthy volunteers received a broad neuropsychological battery that covered multiple cognitive domains including measures of premorbid IQ, verbal and nonverbal IQ, memory, and executive function. The latter two domains were included given evidence that patients may show poor awareness of cognitive functioning in these domains [25,26]. For each cognitive measure, age-corrected *z*-scores were computed based on the normative data from test manuals. The cognitive domains and specific measures that were used included:

*Premorbid IQ: Reading*: North American Adult Reading Test estimated full scale IQ score [35].

*Verbal IQ: Verbal Knowledge/Vocabulary*: Kaufman Brief Intelligence Test-2 (K-BIT-2) [36] Verbal Knowledge subtest.

*Verbal IQ: Verbal Comprehension/Reasoning*: K-BIT-2 Riddles subtest.

*Nonverbal IQ: Nonverbal Reasoning*: K-BIT-2 Matrices subtest.

*Verbal Memory*: Due to participant’s involvement in a different study, we used a modified version of the Rey Auditory Verbal Learning Test (RAVLT) [37] which only involved the presentation of learning Trials 1–3 recall, rather than Trials 1–5 as is customary. The use of three learning trials was deemed appropriate, however, as significant learning deficits have been detected in patients with bipolar disorder even on simpler verbal list learning memory tasks that involve three learning trials (e.g., Hopkins Verbal Learning Test) [38,39]. In addition, normative data were available to calculate learning across 3 learning trials of the RAVLT [40].

*Nonverbal Memory*: The Extended Complex Figure Test (ECFT) [41] involves an initial copy trial where the participant is shown a visual stimulus and asked to copy it as accurately as possible. This is followed by a 30-min delayed free recall trial where the participant is asked to recall the visual design from memory. The memory score employed was the delayed free recall Score.

*Executive: Visual Construction*: To assess the visual organization/construction aspect of executive function, the ECFT Copy Trial score was used.

*Executive: Attentional Shifting*: Trailmaking Test A and B time to completion [42]. Despite the fact that Trails A primarily involves psychomotor processing speed and Trails B adds an executive attentional shifting component, these two trials are also quite similar. We elected to use the mean Trails A and B performance as the primary score in this study for the following reasons: (a) prior work shows that these two scores are highly correlated [40], and in our prior metacognitive study we also observed a strong correlation of (*r* = 0.57) between Trails A and B [15] and (b) previous neuropsychological factor analytic studies in bipolar disorder have demonstrated that Trails A and B load on a common cognitive factor [43,44].

A global cognitive score was calculated by averaging the performance on all eight measures presented above.

#### Specific metacognitive measures

For each of the cognitive measures above, just prior to starting a task, participants were asked to predict how well they thought they would perform on the task using a Likert scale as follows: Compared to healthy people my age, I believe that on the upcoming ____________ test my performance will be: (−3) profoundly below average, (−2) well below average, (−1) below average, (0) average, (1) above average, (2) well above average, and (3) superior. The specific wording that was used to describe each task (i.e., which occupied the blank in the previous sentence) was as follows to reflect each of the different tasks: word pronunciation, verbal knowledge and vocabulary, riddles, visual problem solving, verbal memory, visual memory, design copy, and connect-the-dots.

The Likert rating was purposely scaled to correspond with the *z*-score reflecting performance on the respective cognitive task. For example, a Likert rating scale score of −1 indicated that the participant rated their ability to be below average relative to same-age peers. Similarly, a *z*-score of −1 on the cognitive task indicated low average cognitive task performance relative to same-age peers. This parallel scaling allowed for the comparison of ratings to performance through the generation of difference scores [15,45–47], described below.

In addition to the prediction rating, a postdiction rating was obtained for each task immediately after its completion using the same Likert scale: Compared to healthy people my age, my ability to ___________________ on the previous test was: (−3) to (3) as above. The specific wording to describe each task (which occupied the blank in the previous sentence) was as follows: pronounce words, verbal knowledge and vocabulary, solve riddles, solve visual problems, remember words, remember a design, accurately copy a design, and rapidly connect dots.

The five primary metacognitive measures (for each task) employed in this study consisted of MK and ME signed difference scores (SDS), MK and ME unsigned difference scores (UDS), and a change score (CS) as described below. Data supporting acceptable test–retest reliabilities for these types of measures is reported elsewhere [15].

##### Metacognitive knowledge signed difference score (MKSDS)

The MKSDS score, which measures absolute accuracy of ratings, was derived by subtracting the participant’s actual test performance *z*-score from their prediction rating. Accordingly, an MKSDS score of 0 indicates that there was no discrepancy between the individual’s performance and their rating of performance, reflecting accurate awareness of their ability. In contrast, higher positive values reflect increasing overestimation of ability, whereas lower (more negative) values reflect increasing underestimation of ability. This score relies on metacognitive knowledge because in order to come up with a prediction, the individual is drawing largely on their pre-existing knowledge or judgment of their ability in the given cognitive area. Similar measures have been employed previously in bipolar disorder [15,26,27].

##### Metacognitive knowledge unsigned difference score (MKUDS)

The MKUDS was calculated by taking the absolute value of the MKSDS. This value provides an index of nondirectional absolute accuracy of the rating—that is, regardless of whether the individual is over- or under-estimating [14,15,48,49]. Thus, values of zero represent maximal accuracy, and increasing values reflect decreasing accuracy.

##### Metacognitive experience signed difference score (MESDS)

This score was calculated by subtracting the test performance *z*-score from the postdiction rating. This score relies on metacognitive experience because in order to come up with the postdiction rating, the individual is drawing largely on their recent experience with the task.

##### Metacognitive experience unsigned difference score (MEUDS)

This value was derived by taking the absolute value of the MESDS, where zero represents maximal accuracy and increasing values reflect decreasing accuracy.

##### Change score (CS)

CS was calculated by subtracting each subject’s prediction score from their postdiction score, and reflects the degree to which the individual’s subjective ratings change after exposure to the task. Scores of zero reflect no change, increasing positive scores reflect perceived improvement in performance, and negative scores reflect perceived decline in performance.

### Global metacognitive measures

As in our previous study [15], prior to all cognitive testing, participants were asked to provide a global rating about their perceived cognitive functioning in the following manner: “Compared to healthy people my age, I believe that my cognitive skills (concentration, memory, problem solving) are ______.” When this rating was contrasted to their global cognitive score, it was possible to calculate MKSDS and MKUDS scores for global cognitive functioning.

### Quality of life (QoL)

QoL was assessed using the Quality of Life in Bipolar Disorder (QoL.BD) scale, which was developed specifically to evaluate QoL in individuals living with bipolar disorder across a range of 14 life domains [50]. The scale requires responses on individual items using a 5-point likert scale, with higher scores reflecting subjectively higher QoL. We used the total score based on the sum of items across the 12 primary domains (4 questions per domain), which yields scores ranging from a minimum of 48 to a maximum of 240.

### Statistical analysis

Statistical analyses were conducted using SPSS 25.0 (SPSS Inc., Chicago, IL). Based on our prior metacognitive study in bipolar disorder, we were able to detect a medium-sized effect in MK SDS scores when comparing patients to controls [15]. Thus, in the current study, we determined that a sample size of approximately 51 participants per group would be required to detect a medium sized effect (0.5 Cohen’s *d* effect size) with 0.80 power (one-tailed).

All cognitive performance scores and all metacognitive measures were checked for normality of distribution using Shapiro–Wilk normality tests. Demographic comparisons between groups were conducted using *t*-tests or chi-square statistics as appropriate. *t*-tests or Mann–Whitney *U* tests were used to assess group differences in all cognitive performance scores and all metacognitive measures. Although this study was sufficiently powered to detect meaningful effects, the sample size in this study was nevertheless modest and there was some variation in sample sizes across different metacognitive measures. Because significance levels (*p*-values) are highly sensitive to sample size variation, we chose to preferentially focus on the magnitude of effects rather than to correct *p*-values for multiple comparisons, especially in light of the recognized limitations of relying on significance levels [51]. Based on our prior work [15], the effect size magnitude for global metacognitive impairment that we observed was approximately *d* = .44. Therefore, with guidance from this prior work, in the interpretation of our results we considered effect sizes with magnitudes of *d* = 0.4 or stronger to represent meaningful effects. In order to evaluate whether there were differences in SDS scores across cognitive domains, we used profile analysis, testing for flatness using Hotelling’s criterion. This was done separately for patients and controls, for MK and ME tasks. Spearman correlations were used to evaluate the relationship between metacognitive measures and clinical variables and QoL.

## Results

### Sample demographics and clinical variables

Table 1 summarizes demographic variables in the bipolar (*n* = 57) and control (*n* = 55) groups, as well as clinical variables for the bipolar sample. The bipolar disorder sample consisted of 45 individuals with a primary diagnosis of bipolar I (78.9%) and 12 with bipolar II (21.1%). Participants with bipolar disorder had a mean age of 37.1 (SD = 10.1) years, and a duration of illness of 19.9 (SD = 11.3) years. Groups were comparable with regard to all primary demographics including age, education, premorbid IQ, ethnicity, and sex (Table 1). Due to a delay in initiation of some of the metacognitive tasks in the control group, the sample sizes were uneven across tasks for controls (see Table 2).

**Table 1.** Demographic and clinical characteristics for individuals with bipolar disorder and healthy participants.

	Bipolar disorder (*n* = 57)		Healthy volunteers (*n* = 55)		Group difference statistics	
Continuous variable	*M*	SD	*M*	SD	*t*	*p*
Age	37.09	10.12	37.05	10.58	0.02	0.99
Education	15.28	2.08	15.65	1.92	−0.99	0.33
Premorbid IQ (NAART)	108.98	7.92	107.77	7.76	0.82	0.42
Duration of illness, years	19.93	11.32	–	–	–	–
Number of hospitalizations^a^	2.99	3.35	–	–	–	–
Age at mania/hypomania onset	21.06	6.86	–	–	–	–
Age at depression onset^b^	17.77	6.39	–	–	–	–
YMRS	1.42	2.15	–	–	–	–
HAMD	3.26	2.32	–	–	–	–
QoL-BD	173.9	21.1				
Categorical variable	*N*	%	*N*	%	*X^2^*	*p*
Gender					.003	0.96
Males	21	36.8	20	36.4		
Ethnicity					1.95	0.16
Caucasian	45	78.9	37	67.3		
Employment status					3.48	0.18
Employed at least half-time	27	47.4	34	61.8		
Student at least half-time	6	10.5	7	12.7		
Neither	24	42.1	14	25.5		
Medications						
Mood stabilizers	49	86				
Lithium	24	42.1				
Valproate	20	35.1				
Lamotrigine	18	31.6				
Antipsychotics	31	54.4				
Antidepressants	27	47.4				
History of alcohol/substance abuse	36	63.2				
History of depression	55	96.5				
History of psychosis	29	50.9				

Abbreviations: HAMD, Hamilton Depression rating scale; NAART, North American Adult Reading Test; QoL-BD, Quality of Life—Bipolar Disorders; YMRS, Young Mania Rating Scale.

a Based on *n* = 56.

b Based on *n* = 55.

**Table 2.** Summary of group differences in objective cognitive performance.

Cognitive domain	Bipolar participants (*n* = 57)	Healthy volunteers (*n* = 55)	*t*-test		Mann–Whitney		Cohen’s *d*
			*t*	*p*	*z*	*p*	
Reading	0.60 ± 0.53	0.52 ± 0.52			−0.760	0.447	0.15
Verbal knowledge	0.36 ± 0.70	0.10 ± 0.75			−1.689	0.091	0.36
Verbal reasoning	−0.02 ± 0.81	0.03 ± 0.91			−0.669	0.503	−0.06
Nonverbal reasoning	0.47 ± 0.92	0.56 ± 0.80	−0.568	0.571			−0.10
Verbal memory	0.60 ± 1.13	0.53 ± 0.95	0.334	0.739			0.07
Nonverbal memory	0.08 ± 0.93	0.36 ± 0.73			−1.675	0.094	−0.33
Construction	0.87 ± 0.87	1.17 ± 0.60			−1.785	0.074	**−0.40**
Attentional shifting	−0.16 ± 1.02	0.02 ± 0.92	−0.951	0.344			−0.19
Trails A	0.02 ± 1.15	0.10 ± 1.14	−0.361	0.719			−0.07
Trails B	−0.35 ± 1.14	−0.07 ± 1.00	−1.337	0.184			−0.26
Global cognition	0.35 ± 0.57	0.40 ± 0.44	−0.487	0.627			−0.10

Effect sizes of magnitude 0.40 or stronger are bolded.

For Reading, Verbal Knowledge, Verbal Reasoning, Nonverbal Reasoning, and Attentional Shifting, control *n* = 48; for Nonverbal Memory, Construction, control *n* = 45.

* *p* < 0.05.

** *p* < 0.01.

### Group differences in cognitive performance

Table 2 presents a summary of objective cognitive test performances in individuals with bipolar disorder and healthy volunteers. Participants with bipolar disorder showed numerically lower performances on most measures than the comparison group, although none of the differences reached statistical significance (*p* > .05). Nevertheless, the effect size for the construction task was of moderate magnitude (*d* = 40), favoring controls over patients.

### Group differences in primary metacognitive variables

Table 3 summarizes the results for the primary metacognitive measures, and results below focus on those measures showing patient-control effect size magnitudes of 0.40 or stronger.

**Table 3.** Summary of group differences in metacognitive variables.

Cognitive measure	Bipolar participants (*n* = 57)	Healthy volunteers (*n* = 55)	*t*-test		Mann–Whitney		Cohen’s *d*
			*t*	*p*	*z*	*p*	
Reading							
MKSDS	0.03 ± 1.22	0.16 ± 0.90	−0.620	0.537			−0.12
MKUDS	0.88 ± 0.83	0.73 ± 0.55			−0.412	0.681	0.21
MESDS	−0.16 ± 1.03	−0.17 ± 0.84	0.047	0.963			0.01
MEUDS	0.84 ± 0.60	0.61 ± 0.60			−2.297	0.022*	0.38
Change	0.19 ± 1.03	0.33 ± 0.78			−0.555	0.579	−0.15
Verbal knowledge							
MKSDS	0.08 ± 1.02	0.51 ± 0.78			−2.048	0.041*	**−0.47**
MKUDS	0.73 ± 0.71	0.72 ± 0.59			−0.255	0.798	0.02
MESDS	−0.06 ± 1.11	−0.01 ± 0.85	−0.257	0.798			−0.05
MEUDS	0.85 ± 0.72	0.63 ± 0.57			−1.554	0.120	0.34
Change	0.14 ± 0.88	0.52 ± 0.65			−2.468	0.014*	**−0.49**
Verbal reasoning							
MKSDS	−0.61 ± 1.10	−0.38 ± 1.27	−0.978	0.330			−0.19
MKUDS	1.01 ± 0.75	0.98 ± 0.88			−0.487	0.626	0.04
MESDS	−0.03 ± 1.05	0.01 ± 1.00	−0.215	0.830			−0.04
MEUDS	0.77 ± 0.70	0.76 ± 0.64			−0.213	0.832	0.01
Change	−0.58 ± 1.28	−0.40 ± 0.79			−0.559	0.576	−0.17
Nonverbal reasoning							
MKSDS	−0.47 ± 1.11	−0.23 ± 0.83	−1.246	0.216			−0.24
MKUDS	0.95 ± 0.74	0.67 ± 0.53			−1.776	0.076	**0.44**
MESDS	−0.78 ± 1.16	−0.38 ± 0.80	−2.051	0.043*			**−0.40**
MEUDS	1.13 ± 0.83	0.66 ± 0.58			−3.129	0.002**	**0.66**
Change	0.32 ± 1.09	0.15 ± 0.71			−0.985	0.324	0.18
Verbal memory							
MKSDS	−1.05 ± 1.35	−0.50 ± 1.20	−2.306	0.023*			**−0.43**
MKUDS	1.33 ± 1.07	1.06 ± 0.74			−0.981	0.327	0.29
MESDS	−0.91 ± 1.27	−0.60 ± 0.87	−1.506	0.135			−0.28
MEUDS	1.26 ± 0.92	0.84 ± 0.64			−2.340	0.019*	**0.53**
Change	−0.14 ± 1.01	0.11 ± 0.96			−1.089	0.276	−0.25
Nonverbal memory							
MKSDS	−0.25 ± 1.26	−0.16 ± 1.03	−0.413	0.681			−0.08
MKUDS	0.98 ± 0.82	0.78 ± 0.69			−1.195	0.232	0.26
MESDS	−0.73 ± 1.18	−0.84 ± 0.89			−0.095	0.924	0.11
MEUDS	1.09 ± 0.85	0.96 ± 0.76			−0.760	0.448	0.16
Change	0.47 ± 1.24	0.69 ± 1.08			−0.828	0.408	−0.19
Construction							
MKSDS	−0.66 ± 1.19	−0.66 ± 0.87			−0.051	0.959	0.00
MKUDS	1.06 ± 0.85	0.88 ± 0.63			−0.872	0.383	0.24
MESDS	−0.29 ± 1.16	−0.64 ± 0.88			−1.677	0.094	0.34
MEUDS	0.92 ± 0.75	0.93 ± 0.54			−0.623	0.533	−0.02
Change	−0.37 ± 1.10	−0.02 ± 0.69			−1.721	0.085	−0.38
Attentional shifting							
MKSDS	0.16 ± 1.39	0.42 ± 1.10	−1.032	0.304			−0.21
MKUDS	1.07 ± 0.90	0.96 ± 0.66			−0.203	0.839	0.14
MESDS	0.08 ± 1.28	0.34 ± 1.02	−1.134	0.259			−0.22
MEUDS	1.00 ± 0.79	0.90 ± 0.59			−0.267	0.789	0.14
Change	0.09 ± 1.02	0.08 ± 0.92			−0.122	0.903	0.01
Global cognition							
MKSDS	0.14 ± 1.17	0.55 ± 0.78	−2.169	0.032*			**−0.41**
MKUDS	0.88 ± 0.78	0.74 ± 0.60			−0.597	0.551	0.20

For Reading, Verbal Knowledge, Verbal Reasoning, Nonverbal Reasoning, and Attentional Shifting, control *n* = 48; for Nonverbal Memory, Construction, control *n* = 45.

Abbreviations: MESDS, Metacognitive Experience Signed Difference Score; MEUDS, Metacognitive Experience Unsigned Difference Score; MKSDS, Metacognitive Knowledge Signed Difference Score; MKUDS, Metacognitive Knowledge Unsigned Difference Score. Effect sizes of magnitude 0.40 or stronger are bolded.

* *p* < 0.05.

** *p* < 0.01.

**Figure 1.**
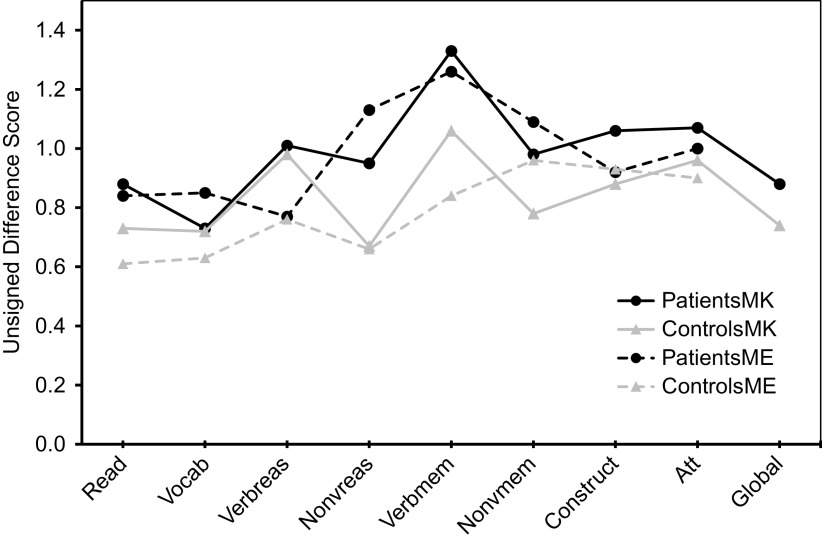
Accuracy of metacognitive knowledge and experience ratings (unsigned difference scores) across multiple cognitive domains in participants with bipolar disorder and healthy volunteers. Higher values indicate more inaccurate ratings.

**Figure 2.**
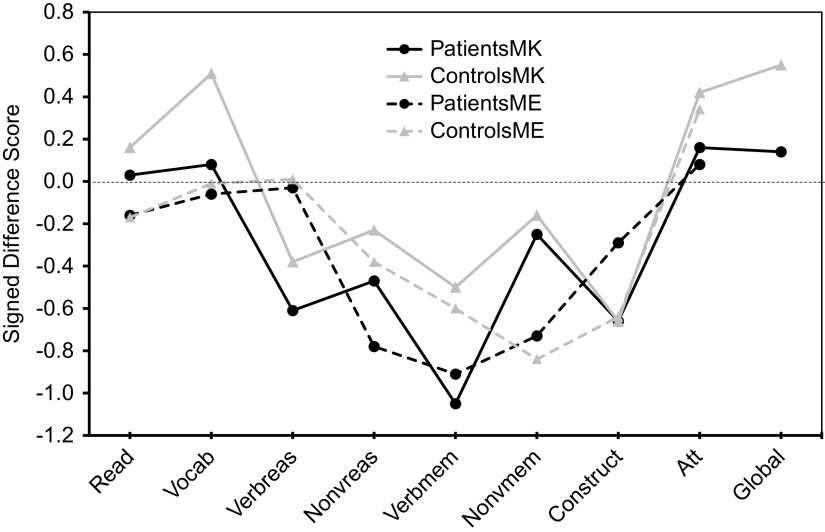
Accuracy of metacognitive knowledge and metacognitive experience ratings (signed difference scores) across multiple cognitive domains in participants with bipolar disorder and healthy volunteers. Positive values indicate overestimation, and negative values indicate underestimation.

Regarding absolute accuracy regardless of over- or under-estimation (UDS scores), individuals with bipolar disorder were less accurate in their MK ratings for nonverbal reasoning (*Z* = −1.78, *p* = 0.08; *d* = 0.44) and in their ME ratings for nonverbal reasoning (*Z* = −3.13, *p* = 0.002; *d* = 0.66) and verbal memory (*Z* = −2.34, *p* = 0.02; *d* = 0.53) than healthy participants (Figure 1). With regard to the directional measures of metacognitive accuracy (SDS scores), those with bipolar disorder showed MK underestimation in verbal knowledge/vocabulary (*Z* = −2.05, *p* = 0.04; *d* = −0.47), verbal memory [*t*(110) = −2.31, *p* = 0.02; *d* = −0.43], and global cognitive function [*t*(97.87) = −2.17, *p* = 0.03; *d* = −0.41], and ME underestimation in nonverbal reasoning [*t*(103) = −2.05, *p* = 0.04; *d* = −0.40] relative to controls (Figure 2).

### SDS scores across cognitive domains

Profile analysis revealed that there was a significant difference in SDS scores across cognitive domains for MK in patients [*F*(8, 49) = 11.7, *p* < 0.001)] and controls [*F*(8, 37) = 18.4, *p* < 0.001)], as well as for ME in patients [*F*(7, 50) = 5.0, *p* < 0.001)] and controls [*F*(7, 38) = 7.5, *p* < 0.001)]. As illustrated in Figure 2, the bipolar group’s MK underestimation for verbal memory and their ME underestimation for nonverbal reasoning reflected even stronger underestimation than controls, as controls also underestimated their performance because their scores on these measures also fell below the “zero” line that indicates perfect accuracy. In contrast, Figure 2 shows that MK underestimations in bipolar disorder for verbal knowledge/vocabulary and for global cognition were actually more accurate in absolute terms, as their SDS scores were closer to the “zero” line than controls. Thus, in these instances bipolar participant’s underestimations could more aptly be viewed as control participant overestimations of ability that were absent in individuals with bipolar disorder.

### Group differences in change scores

Regarding change scores (Table 3), the only significant difference between participants with bipolar disorder and controls was observed for verbal knowledge/vocabulary [*t*(103) = −2.49, *p* = 0.02). In this case ratings decreased more for controls than for individuals with bipolar disorder after being exposed to the task.

### Correlation between metacognitive scores and clinical variables and QoL

The association between metacognitive variables and clinical variables and QoL are presented in Table 4.

**Table 4.** Correlations between metacognitive variables and clinical characteristics and quality of life.

	Clinical variables				
Cognitive domain	Mood stabilizer	Antipsychotic	HAMD	YMRS	QoL-BD
Reading					
MKSDS	0.06	−0.19	0.15	0.05	0.06
MKUDS	−0.02	−0.15	0.08	0.11	−0.03
MESDS	0.21	−0.10	0.08	−0.02	−0.05
MEUDS	−0.01	−0.06	−0.37**	−0.07	0.14
Change	−0.09	−0.04	0.10	0.09	0.06
Verbal knowledge					
MKSDS	−0.01	−0.08	−0.04	−0.14	0.11
MKUDS	0.09	−0.29*	0.04	0.16	−0.06
MESDS	0.07	0.02	0.19	−0.06	−0.20
MEUDS	−0.17	−0.17	0.09	0.12	−0.19
Change	−0.11	−0.05	−0.20	−0.03	0.36**
Verbal reasoning					
MKSDS	0.06	−0.08	0.03	0.01	0.10
MKUDS	−0.01	−0.11	0.18	0.21	0.02
MESDS	0.02	−0.07	0.23	0.10	−0.09
MEUDS	0.01	−0.11	−0.03	0.17	0.07
Change	0.06	−0.04	−0.13	−0.06	0.11
Nonverbal reasoning					
MKSDS	0.07	−0.31*	0.21	0.32*	0.11
MKUDS	−0.12	0.21	−0.09	0.02	0.00
MESDS	0.18	−0.14	0.08	0.06	0.05
MEUDS	−0.04	0.04	0.07	−0.03	−0.26
Change	−0.19	−0.14	0.01	0.19	0.11
Verbal memory					
MKSDS	0.00	−0.18	0.19	0.18	0.00
MKUDS	0.24	0.07	−0.12	−0.16	−0.02
MESDS	0.13	−0.24	0.11	0.07	0.07
MEUDS	0.21	0.03	−0.09	−0.01	−0.09
Change	−0.27*	0.10	0.09	0.15	−0.01
Nonverbal memory					
MKSDS	0.08	−0.29*	−0.11	0.18	0.16
MKUDS	0.16	−0.09	0.21	−0.08	−0.27*
MESDS	0.05	−0.05	−0.04	−0.10	0.15
MEUDS	0.19	−0.07	0.21	0.27*	−0.25
Change	−0.13	−0.14	−0.17	0.22	0.13
Construction					
MKSDS	−0.14	−0.07	−0.04	0.19	0.33*
MKUDS	0.21	0.03	0.14	−0.15	−0.42**
MESDS	−0.26*	−0.06	0.10	0.33*	0.07
MEUDS	0.15	−0.20	0.10	−0.13	−0.05
Change	0.21	0.06	−0.16	−0.09	0.27*
Attentional shifting					
MKSDS	−0.03	0.03	0.01	0.18	0.21
MKUDS	−0.18	−0.05	0.01	0.18	−0.04
MESDS	0.11	−0.02	0.01	−0.14	−0.06
MEUDS	−0.11	0.09	0.00	0.07	−0.01
Change	−0.15	0.07	−0.02	0.38**	0.27*
Global cognition					
MKSDS	0.12	0.00	0.08	0.08	0.21
MKUDS	0.21	−0.22	0.14	0.15	−0.08

Exposure to medications was coded as either 1 = yes, or 2 = no for both mood stabilizers and antipsychotics

Data are Spearman correlation coefficients.

Abbreviations: HAMD, Hamilton Depression Rating Scale Score; MESDS, Metacognitive Experience Signed Difference Score; MEUDS, Metacognitive Experience Unsigned Difference Score; MKSDS, Metacognitive Knowledge Signed Difference Score; MKUDS, Metacognitive Knowledge Unsigned Difference Score; QoL-BD, Quality of Life—Bipolar Disorders Scale; YMRS, Young Mania Rating Scale Score.

* *p* < 0.05.

** *p* < 0.01.

Treatment with either antipsychotics or mood stabilizers was minimally associated with metacognitive measures. Similarly, there were few significant correlations between symptom ratings of depression or mania and metacognitive measures. For a very limited number of variables (MKSDS nonverbal reasoning and MESDS construction), increasing subsyndromal hypomanic symptoms were modestly associated with increasing self-rating of cognition (*r* = 0.32, *r* = 0.33, respectively). In contrast, there were more significant correlations between metacognitive variables and QoL. Increased MK inaccuracy was associated with poorer QoL for nonverbal memory (*r* = −0.27) and construction (*r* = −0.42); moreover, for construction, increasing MK self-ratings were associated with better QoL (*r* = 0.33). QoL was also associated with change scores for verbal knowledge/vocabulary, construction, and attentional shifting, such that increased perceived performance following these tasks was related to better QoL. (*r* = 0.36, *r* = 0.27, *r* = 0.27, respectively).

For all the significant correlations between metacognitive variables and QoL presented in Table 4, we also ran partial correlations controlling for antipsychotic use and depressive and manic symptoms. With the exception of one of these metacognitive variables, all the remaining correlations remained significant (and one correlation became a trend). Thus, findings could generally not be attributed to mood symptoms or antipsychotic use.

## Discussion

### General findings

A primary finding of this study was that for most cognitive domains (5 of 8), participants with bipolar disorder showed comparable metacognitive accuracy relative to control participants, both in MK and ME. However, for three cognitive domains (verbal knowledge/vocabulary, verbal memory, and nonverbal reasoning) and for global cognitive functioning, individuals with bipolar disorder showed some level of metacognitive inaccuracy (i.e., deviation from that observed in the control group). These metacognitive deviations occurred in both MK and ME and more often reflected underestimation of cognitive functioning relative to control participants, although in some instances reflected both under- and over-estimation. Refer to Table 5 for a summary of the main findings of this study.

**Table 5.** Summary of impairments in objective cognition and metacognition in bipolar disorder.

Cognitive domain	Objective cognition	Metacognitive knowledge	Metacognitive experience	Metacognitive change
Reading				
Verbal knowledge		x (u)		x
Verbal reasoning				
Nonverbal reasoning		x	x (u)	
Verbal memory		x (u)	x	
Nonverbal memory				
Executive—Construction	x			
Executive—Attentional shifting				
Global cognition		x (u)		

Abbreviation: (u) = underestimation.

The finding of metacognitive impairment in verbal memory was consistent with our hypothesis and with prior work [26]; however, we failed to observe a hypothesized metacognitive deficit in the executive functioning measures. The negative executive finding suggests that patients may show intact metacognition even in cognitive domains where they often show cognitive deficits. Alternatively, the negative executive findings may be due to the fact that we only assessed attentional shifting and visual organization components of executive function, and that patients may show metacognitive deficits in other executive components such as working memory or inhibition. Notably, individuals with bipolar disorder also showed metacognitive deficits in some domains that are typically preserved in bipolar disorder, such as vocabulary/verbal intellectual abilities [1,2]. Thus, patients may exhibit metacognitive deficits in domains regardless of whether they show cognitive deficits in those areas. Overall, given the disparity of metacognitive inaccuracies across different cognitive domains, our results are broadly consistent with domain-specific accounts of human metacognition [13,52,53].

### MK and ME findings

Aberrations in MK (i.e., ability to predict performance) in participants with bipolar disorder were observed in the areas of verbal knowledge/vocabulary, nonverbal reasoning, and verbal memory. For verbal knowledge/vocabulary and verbal memory, inaccuracies involved underestimation of ability; however, for nonverbal reasoning inaccuracies reflected both over- and under-estimation. MK impairment (underestimation) was also evident on the global prediction rating in individuals with bipolar disorder, consistent with our prior findings in a different patient sample [15]. Together, these MK findings indicate that patient’s general beliefs about their cognitive functioning in these specific domains and in their global cognitive functioning are inaccurate. In turn, these metacognitive inaccuracies may account for the frequent observation of poor concordance between subjective ratings of cognitive function and objective cognitive performance in bipolar disorder [19,25].

In addition to MK impairments, individuals with bipolar disorder showed poor monitoring of the accuracy of their recent, online performance in several cognitive domains (impaired ME). These deficits occurred in nonverbal reasoning and verbal memory, with the former reflecting underestimation of ability. The observation of deficits in both MK and ME represents a key finding of this study, as prior studies in this population were limited by either only assessing global cognitive functions or by only assessing metacognitive accuracy in a single specific cognitive domain [15,27].

The finding that the majority of metacognitive inaccuracies reflected underestimation of abilities is consistent with proposals that individuals with bipolar disorder, even in the euthymic state, may show bias toward negative information processing, exhibit impaired cognitive control of emotional material, or engage in emotion-regulation strategies such as dampening of positive affect [54–56]. These altered underlying processes may contribute to individual’s tendencies to view some of their cognitive abilities in a negative light, resulting in underestimation of their abilities. For some abilities (predictions of verbal memory and postdictions of nonverbal reasoning), control participants also underestimated their abilities; however, the magnitude of underestimation in persons with bipolar disorder was more pronounced. For other abilities (prediction of verbal knowledge/vocabulary and global cognitive function), patient’s underestimations reflected an absence of overestimation that was present in control participants. The tendency to overestimate skills and abilities in nonclinical populations has been well documented [57,58], and may represent an adaptive cognitive bias or coping mechanism aimed at preserving positive affect or self-esteem, and enhancing well-being [59,60]. The present data demonstrate that in some instances, people with bipolar disorder may lack this adaptive bias to view their skills in a positive light, resulting in a more “realistic” appraisal of their ability, akin to the depressive realism that has been described in depression [61].

The tendency to view one’s ability in a positive light, however, was not universal across all cognitive domains even in controls, as metacognitive accuracies varied significantly across different cognitive domains, as observed in prior studies [14,62]. Specifically, Figure 2 reveals that in all groups, abilities such as nonverbal reasoning and verbal and nonverbal memory tended to be underestimated more than other abilities such as word pronunciation/reading, verbal knowledge/vocabulary, attention, and global cognition. Miskowiak et al. [26] also reported a tendency for individuals with remitted bipolar disorder to overestimate their attention skills in comparison to their memory skills. It is noteworthy that the observed underestimation in bipolar participants in the present study occurred in cognitive domains where healthy participants both over- and under-estimated their own ability, which speaks to the robustness of the relative underestimation in the bipolar sample. Moreover, because individuals with bipolar disorder were euthymic and because mood ratings were generally not associated with metacognitive variables, it can be inferred, although not conclusively, that metacognitive impairments such as a potential bias toward negative cognitive self-appraisal is more likely to be a trait feature of the illness.

### Change in ratings as a result of task exposure

A further goal of the present study was to evaluate the degree to which exposure to a cognitive task might influence metacognitive ratings and accuracy. For most cognitive domains, task exposure had a similar impact on ratings in both groups. However, for verbal knowledge/vocabulary, control participant overestimations were reduced to more accurate ranges (i.e., close to zero), whereas in bipolar disorder ratings remained relatively accurate and unchanged in response to experiencing the task. It is possible that this pattern resulted from the absence of a positive bias in bipolar disorder at the time the prediction was made, and this pattern remained consistent after task exposure. These data suggest that in some circumstances individuals with bipolar disorder may exhibit more resistance to modify self-perceptions in the face of experience with a task, consistent with cognitive inflexibility [63,64].

### Correlates of metacognitive measures

The analysis of the association between metacognitive and clinical variables provided little support for the idea that metacognitive inaccuracies were related to either subsyndromal mood symptoms or medication variables (Table 4). However, a greater number of significant correlations were evident between metacognition variables and QoL, and this could not be attributed to mood symptoms or antipsychotic use. For several cognitive domains, increased metacognitive inaccuracies were associated with reduced QoL. Additionally, for verbal knowledge/vocabulary, visual construction, and attentional shifting, individuals who rated that their performance had improved after completing the tasks showed higher ratings of QoL. This finding may suggest that an individual’s flexibility, willingness, or ability to change ratings in a positive direction, thus challenging or modifying negative biases, may be associated with better well-being. These findings are consistent with another report showing that remitted participants with bipolar disorder who underestimate their cognitive functioning showed lower socio-occupational functioning and QoL [26].

### Limitations

The findings from this study need to be evaluated within the context of several study limitations. Most significantly, both the patient and control groups in this study were on average highly educated. Therefore, elevated cognitive reserve in patients may have contributed to the observation that even though participants with bipolar disorder performed more poorly numerically than controls in most cognitive domains, the differences did not reach statistical significance (although the effect size for visual construction impairment was moderate in magnitude). Thus, our study sample may not be entirely representative of the broader population of individuals with bipolar disorder, and replication and further study in less educated samples is warranted. Nevertheless, considering that self-ratings of ability may be influenced by differences in levels of cognitive performance between groups [58], the metacognitive group differences observed herein may be viewed as particularly robust given the relative absence of significant cognitive differences between our two groups. Another possible limitation relates to the fact that in our methodology we combined the Trails A and B tasks, based on their strong correlation, to derive a composite score of executive attentional shifting ability; however, this may have diluted the precision of measurement of this executive component, potentially influencing findings. Therefore, in future studies it may be more prudent to have subjects rate executive attentional shifting before and after Trails B exclusively, rather than before and after completing both Trails A and B. As previously mentioned, another study weakness is that the full range of cognitive and executive skills that are implicated in bipolar disorder were not assessed in this study, and future work should incorporate measures of these abilities. Finally, a comprehensive understanding of metacognition in bipolar disorder requires the use of multiple methodologies beyond the global ratings that were employed in this study. Future work should also include methodologies such as those incorporating item-specific metacognitive measures [6,29] and those based on signal detection theory [65].

### Implications and future directions

Although the clinical or treatment implications of these findings are yet to be fully elucidated and require further study, several key points are highlighted. First, future metacognitive research in clinical populations should clearly incorporate normative comparison groups. As this study reveals, interpretation of patterns of cognitive self-ratings in clinical populations is complicated by the fact that both positive and negative ratings/biases can be normative, healthy, or adaptive [66]. This implies that intervention goals may not necessarily be to improve the absolute accuracy of self-views, but rather to align them with a more personally beneficial or adaptive outlook. Thus, it will be critical to understand metacognitive beliefs in nonclinical individuals, across multiple cognitive domains, in order to provide meaningful referents for the beliefs observed in clinical groups [14,67,68]. Finally, future metacognitive studies in mood disorders should continue to investigate euthymic samples in order to minimize the potential influence of acute or subsyndromal mood symptoms on metacognitive ability. This will in turn increase the likelihood that stable trait-related impairments are being assessed.

With this continued understanding it will also be important to investigate the degree to which metacognitive beliefs may be modifiable, and if so, how. Recent work indicates that postdiction feedback and adaptive training have the potential to modify metacognitive accuracies [69,70]. Other strategies including practice, incentives, and self-reflection may also serve to modify self-perceptions [71]. It is hoped that such strategies or interventions can eventually be utilized to improve functional outcomes and QoL in bipolar disorder.

## Data Availability

The datasets generated during and/or analyzed during the current study are available from the corresponding author on reasonable request.
